# Leiomyoma of the Greater Saphenous Vein Mimicking Inguinal Lymphadenopathy

**DOI:** 10.1155/2013/237391

**Published:** 2013-12-02

**Authors:** Dionysios Dellaportas, Thomas Kotsis, Eleni Carvounis, Lazaros Samanides

**Affiliations:** ^1^2nd Department of Surgery, University Hospital Aretaieion, Attiki, 11528 Athens, Greece; ^2^Department of Pathology, University Hospital Aretaieion, Attiki, 11528 Athens, Greece

## Abstract

*Introduction*. Leiomyoma is a benign vascular tumor affecting the greater saphenous vein rarely. Proper histopathological examination sets the diagnosis after complete and wide surgical excision along with a normal portion of the GSV. *Case Presentation*. A 36-year-old woman was admitted to our hospital complaining of a dull ache on her right groin for the last three months, along with a palpable mass on the mentioned area. An ultrasound (U/S) scan revealed a solid mass measuring 3 × 2 cm. After wide surgical excision, pathological examination revealed a leiomyoma of the GSV. *Discussion*. Benign and mostly malignant tumors arising from the GSV are reported in the literature before. Diagnostic modalities and clinical examination cannot set a correct diagnosis preoperatively due to no specific characteristics of these tumors. Possible sarcomatous behaviour has to be kept in mind when treating tumors arising from a vessel wall and wide surgical excision as negative margins should be attempted.

## 1. Introduction

Leiomyomas of deep soft tissue, other than the retroperitoneum and pelvis, including leiomyomas arising in vessels are rare. Leiomyomas arising from veins may be luminal or attached to the vessel wall. We wish to report a case of leiomyoma of the greater saphenous vein that was attached to the vessel wall and clinically presented as inguinal lymphadenopathy treated successfully in our hospital.

## 2. Case Presentation

**Figure 1:**
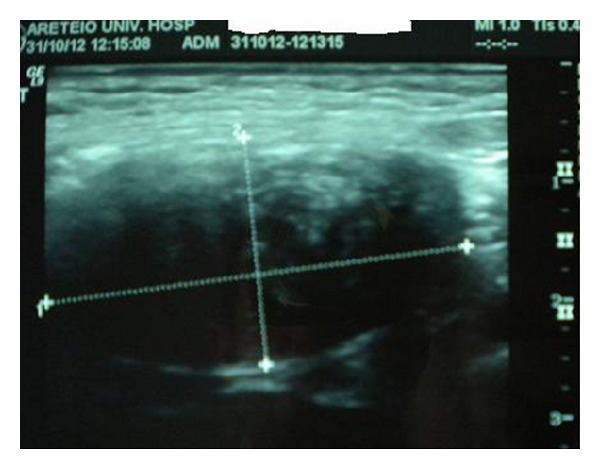
U/S image of the tumor.

**Figure 2:**
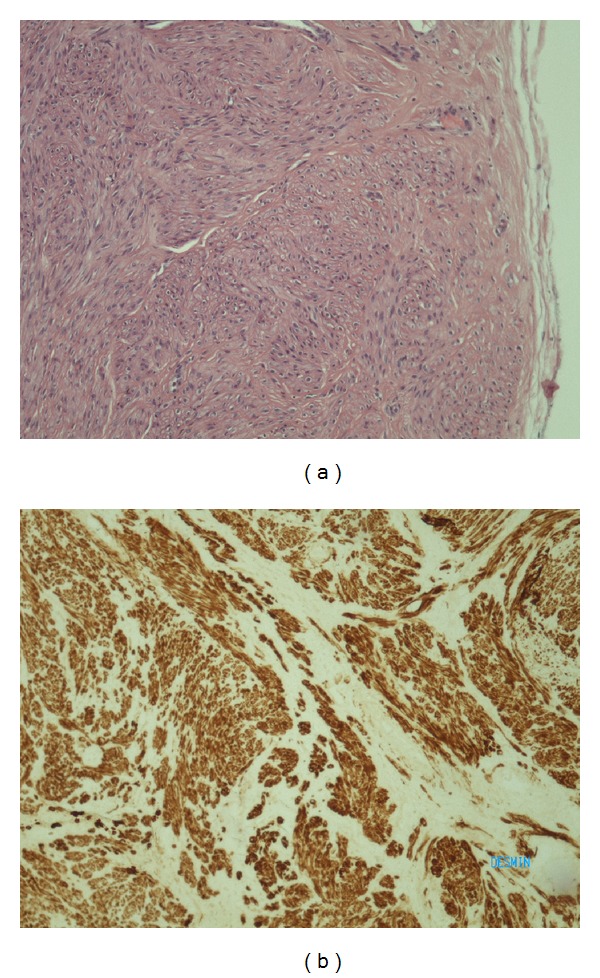
(a) Microscopic image of leiomyoma-sharp margin. (b) Immunohistochemical stain of desmin.

The patient is a 36-year-old woman who was admitted to the hospital complaining of a dull ache in the right inguinal area. On physical examination a firm mass was palpated in that area. Imaging with ultrasound (U/S) scan examination revealed a solid mass measuring 3 × 2 cm (Figure 1). Fine needle aspiration of the mass was inconclusive and complete surgical excision was decided. During surgery it was noted that the tumor was attached to the wall of the greater saphenous vein, and it was removed en bloc with a small part of the vein. The resected specimen was submitted for pathologic evaluation. Grossly the specimen consisted of a firm, well-circumscribed, oval shaped mass measuring 4 × 2 × 1.5 cm. Its cut surface was white in colour and had a whorling pattern reminiscent of leiomyoma. Microscopic examination confirmed the gross impression and proved it to be a smooth muscle tumor (Figure 2(a)). The diagnosis was also confirmed with immunohistochemical stains (desmin +, SMA +, and S-100 protein −) (Figure 2(b)). The tumor also had no areas of necrosis. Its cellularity was moderate and there was no significant nuclear atypia. The mitotic activity was low (up to 1 mitotic figure/40 HPF) and the proliferation index Ki-67 was also low (approximately 1%). Morphologically this tumor was a leiomyoma. The patient was discharged on the first postoperative day.

## 3. Discussion 

Leiomyomas are benign soft tissue tumors that, although very common in other locations, rarely occur in the retroperitoneum, deep soft tissues, and vessel walls. The first case of greater saphenous vein leiomyoma was described by Deweese et al. in 1950 [1], and since then only a few cases are reported in the literature. Usually, these tumors arising from the vessel wall are leiomyosarcomas [2–4]. Clinical examination and common diagnostic imaging modalities cannot set a correct diagnosis preoperatively due to the absence of specific characteristics of these tumors, other than the appearance of a solid mass [5]. Clinically the differential diagnosis includes lymphadenopathy, incarcerated or nonreducible hernias, and various soft tissue tumors. In neoplasms of smooth muscle cell origin with benign morphology (“leiomyomas”) arising in the deep soft tissues and vessels caution should be exercised. In particular, tumors with bland uniform nuclei and division activity up to 4 mitotic figures/10HPF should be categorized as tumors of smooth muscle cell of uncertain malignant potential. Such tumors have the potential to recur [6]. It is posted that among imaging modalities, echo duplex and MRI were best suited for diagnosis of venous tumors [7]. Treatment as in all soft tissue neoplasm is complete surgical excision with as possible as wide margins. Benign and mostly malignant tumors arising from the GSV are reported in the literature before, and their management is mostly based on expert's opinion and no established guidelines exist [8]. Follow-up will, however, be required as the only absolute criterion for malignancy is metastatic disease. In leiomyosarcomas adjuvant radiotherapy should be offered to the patient [9], and chemotherapy is reserved for distant metastatic disease with poor results. In benign cases as in our case, follow-up is warranted due to the rarity of the disease and the uncertain recurrence potential of these tumors.
